# Reduction in Epigenetic Age Acceleration Is Related to Empathy in Mothers with Neglectful Caregiving

**DOI:** 10.3390/brainsci11111376

**Published:** 2021-10-21

**Authors:** Silvia Herrero-Roldán, María José Rodrigo, Juan A. Hernández-Cabrera, Colter Mitchell, Maykel López, Julia Alcoba-Florez, Jonah Fisher, Fernanda Espinosa, Inmaculada León

**Affiliations:** 1 Instituto Universitario de Neurociencia, Universidad de La Laguna, 38200 San Cristóbal de La Laguna, Spain; roldansh@gmail.com (S.H.-R.); mjrodri@ull.es (M.J.R.); jhernand@ull.edu.es (J.A.H.-C.); fernandaespinosagonzalez@gmail.com (F.E.); 2 Facultad de Psicología, Universidad de La Laguna, 38071 San Cristóbal de La Laguna, Spain; 3 Survey Research Center, Institute for Social Research, University of Michigan, Ann Arbor, MI 48109, USA; cmsm@umich.edu (C.M.); jazzfish@umich.edu (J.F.); 4 Population Studies Center, Institute for Social Research, University of Michigan, Ann Arbor, MI 48109, USA; 5 A.I. Virtanen Institute for Molecular Sciences, University of Eastern Finland, 70211 Kuopio, Finland; maykel.lopezrodrigues@uef.fi; 6 Servicio de Microbiología, Hospital Universitario N. S. de Candelaria, 38010 San Cristóbal de La Laguna, Spain; juliaalcoba@gmail.com

**Keywords:** DNA methylation, biological clock, epigenetic aging, child neglect, personality traits

## Abstract

DNA methylation clocks are used as molecular estimators of epigenetic age, but with little evidence in mothers and none in neglectful mothering. We investigated differences in epigenetic age acceleration (EAA) and the role of empathy using the PhenoAge clock. We collected saliva samples from mothers with extreme disregard for their child’s needs (50 in the neglect group, NG) and mothers with non-neglectful caregiving (87 in the control group, CG). Mothers completed an empathy scale, along with questionnaires of their own childhood maltreatment, adverse life events and psychiatric disorders. Sociodemographic variables potentially affecting EAA were also measured. The ANCOVA solution showed a significant increase in EAA in the NG compared to the CG, after adjustment for maternal age, number of pregnancies, financial assistance, adverse events, childhood maltreatment and psychiatric disorder. The group interaction effects showed a reduction in EAA for greater empathic concern and for a higher education level both as positive factors, and an increment in EAA for mothers living in a two-parent family as a risk factor, all in the NG. Our findings open the search for protective factors of EAA associated with caregiver behavior to reduce health vulnerabilities and poor social functioning, especially for mothers at risk of maladaptive caregiving.

## 1. Introduction

Neglect is the most common and severe form of child maltreatment, which consists of the caregivers’ failure to provide the child with food, clothing, shelter, medical care, supervision or emotional support [1,2,3]. Being severely neglected in the early years of life disrupts establishing a secure attachment and healthy psychosocial development for the child [4]. Neglect entails cumulative risk for infant mental health and behavioral problems [1] and leads to neurobiological alterations across their life span [5]. In the same line, mothers with neglectful caregiving have also frequently been maltreated in their infancy, often followed by a long-term risk for psychopathology, teenaged pregnancies, obesity, cardiovascular diseases, cognitive delays and educational failures [6,7]. These conditions usually coexist with other stressors such as poverty, social isolation, domestic violence or substance abuse [8], which have demonstrated a negative cumulative effect on health and wellbeing in adulthood [9,10]. There is evidence that most of these life adversities that challenge maternal and adult functioning have been associated with specific epigenetic modifications [11,12,13]. However, the epigenetic features associated with maternal neglect remains a “neglected” topic.

Investigating the epigenetic features of neglectful caregiving, such as epigenetic age, is essential to improve our knowledge of the parental neglect profile and subsequent early childhood diagnosis. Furthermore, identifying possible psychological protective factors of epigenetic aging could substantially impact educational and social policies to promote adequate care of the child and maternal health and wellbeing. We used the notion of a protective factor in the classical sense of individuals’ attributes or characteristics that help them resist or balance the risks to which they are exposed, thus enabling them to successfully manage life stressors and alter the likelihood of adverse outcomes [14]. This notion draws attention to the idea that protective variables should show their positive effects in the presence of risk variables. These variables are more likely to be found in a vulnerable population (such as mothers with neglectful caregiving) and less likely in a normative population (non-neglect control mothers). In this sense, it is essential to know if personality traits such as empathy, which is trainable, can “positively reverse” epigenetic alterations functionally associate with parental neglect, opening the way to more adaptive parenting and positive adult functioning.

Our study seeks to characterize epigenetic age in mothers with neglectful behavior through a biomarker of aging known as the epigenetic clock. Such biological clocks have been developed in the last decade as a molecular estimator of epigenetic (vs. chronological) age. They are based on levels of DNA methylation (DNAm), an epigenetic modification in which a methyl group is added to a cytosine base, most commonly adjacent to a guanine base in regions called CpG sites. Differential levels of DNA methylation at regulatory regions, such as the gene promoter, can affect gene expression [15]. Many DNA sites across the genome are susceptible to methylation by risk exposures, acting as an interface between the genes and the environment [15,16]. The epigenetic clock provides an accurate estimate of biological age across various tissues and exhibits predictable, progressive changes throughout aging [17]. Several epigenetic clocks (i.e., Horvarth, Hannun, Levine) have been developed that enable identifying specific genomic locations most informative of methylation changes and coupled with a mathematical algorithm for estimating the biological age. The biological clock can be calibrated as either “accelerated” or “decelerated” with respect to chronological age. Positive epigenetic age acceleration (EAA) has been associated with many pathologies and age-related functional decline. In contrast, a negative EAA has been associated with younger individuals healthier than their peers at the same chronological age [11].

To our knowledge, no study has used epigenetic clocks to investigate maternal DNAm in the context of neglectful caregiving. To start filling this knowledge gap, our first aim was to examine the differences in epigenetic age acceleration (EAA) between mothers who neglect their children (neglect group, NG) and the mothers with non-neglectful behavior (control group, CG). The abovementioned maternal risk profile associated with neglect coincides with some of the documented adversities affecting DNAm in adult studies. For example, there is evidence that poor living conditions such as low socioeconomic status or low education level can accelerate epigenetic aging [17,18,19]. Moreover, due to the high biological costs in terms of metabolic regulation, oxidative stress, sensitivity to infection and immune cell proliferation, the number of pregnancies was related to increases in Horvath’s DNAmAge in young mothers [20]. In turn, early exposure to toxic caregiving environments involving childhood stress [21], cumulative childhood maltreatment [12], lifetime stress [13], major depression [22] and traumatic stress [12] have been found to accelerate epigenetic aging in adults. In light of this evidence, we hypothesized that mothers in the NG would show positive EAA compared to those in the CG.

To test the age acceleration hypothesis in the NG we used the PhenoAge epigenetic clock as a biomarker that significantly outperforms the first generation of DNAm multi-tissue age estimators for various aging outcomes. These include all-cause mortality, cancers, health span, physical functioning and Alzheimer’s disease, above and beyond what is explained by chronological time [23]. We used saliva as a suitable, comparable and less invasive means of sample collection [24]. The PhenoAge clock is based on age-related DNAm levels at 513 CpG sites, more CpG sites than any other epigenetic clock, to estimate the biological age across multiple tissues and cells. The PhenoAge biomarker not only captures CpGs that display changes with respect to chronological age but also incorporates age-related biochemical measures (e.g., insulin, cholesterol) that account for differences in risk and physiological status among individuals of the same cohort, being very sensitive to variations in the environment [25].

In our second aim, we examined whether variations in empathy, an essential trait for succeeding in intimate relationships and the social world in general, was associated with reduced EAA. Given that mothers with neglectful behaviors usually suffer from various risk factors that are likely to be associated with EAA, it is essential to find protective factors that can be trained and lead to benefits for mothers in the NG. Many studies have focused on the beneficial effects of healthy lifestyles and educative factors [26,27]. However, exploring psychological factors that can help slow epigenetic aging in adults have received little attention. Here, we propose trait empathy, defined as the ability to recognize and understand another person’s thoughts and feelings [28,29,30], as a psychological candidate to test its potential role in moderating/slowing epigenetic aging.

Two sources of evidence can be provided in favor of this personality trait. Empathy is related to the caregiving role since it stems from ancient subcortical circuits (e.g., brainstem and hypothalamic) associated with affective sensitivity, attachment, survival and wellbeing [31]. In addition to this primary function, empathic capacities have a beneficial effect on adult functioning as a tool to form and maintain social bonds between non-kin individuals [32]. Therefore, empathic capacities are crucial for successfully navigating the social world and reducing the chances of suffering social stress, boosting physical and mental health [33].

Based on the previous evidence, we set out the hypothesis of empathy as a protective factor for neglectful caregiving associated with reductions in EAA for mothers in the NG only since this is the group with a risk profile of adverse conditions that would benefit more. We also accounted for potential effects on EAA of other known risk factors of the neglect profile, such as some sociodemographic conditions, own childhood maltreatment, adverse life events and psychiatric disorders. We also aimed to test both the affective (e.g., empathic concern) and cognitive (e.g., perspective-taking) components of empathy to determine their possible distinctive effects on EAA. The relevance of trait empathic concern in neglectful caregiving has been evidenced in its mediation role in the positive relation of volumetric measures in empathy-related brain areas, such as the insula and right inferior frontal gyrus, with mother-child interactive bonding in a play task [34]. Based on this evidence, we propose that the affective component of empathy would be the most strongly involved in decreasing EAA, especially in adverse contexts with neglectful caregiving.

## 2. Materials and Methods

### 2.1. Participants

One hundred thirty-eight mothers (51 NG and 87 CG) were recruited through the same Municipal Social Services and Primary Health Centers. One participant in the NG showed extremely low epigenetic age (more than 3 SD younger) and was eliminated as an outlier (50 NG) for subsequent analyses. All subjects gave their written informed consent for inclusion following the protocol of the Ethical Committee of Investigation of the Canary Islands University Hospital Complex (code: CHUC_2018_63; date of approval: 14 December 2018), under the Code of Ethics of the World Medical Association (Declaration of Helsinki). General inclusion criteria for both groups were being the biological mother of a child under seven years old who had not been placed in foster care at any point in their history nor been born prematurely or suffered perinatal or postnatal medical complications, according to the pediatricians’ reports. Specific inclusion criteria for a mother in the neglect group were a substantiated case of child neglect registered in the last 12 months by Child Protective Services (CPS) according to the reports of the Social Services and complying with all the indicators of the Maltreatment Classification System (MCS) for severe neglect [35] according to the pediatrician of the Primary Health Center in charge of the case. The specific inclusion criteria for the control group were being biological mothers of children having negative scores in all the MCS neglect indicators and the absence of CPS or Preventive Services records for the family.

Mothers in the NG were younger and had a higher number of pregnancies than mothers in the CG, and the target child had a similar mean age in both groups. Moreover, NG mothers were less likely than mothers in the CG to live in two-parent families and more likely to show a lower educational level and to receive financial assistance than those in the CG, whereas mothers in the two groups shared a similar low percentage living in rural areas and had a moderate-high percentage of unemployment (Table 1).

### 2.2. Psychological Measures

The Stressful and Risky Events Inventory was created by combining items from other questionnaires [36,37], according to their relevance to our population. It comprises 16 self-reported negative events (e.g., divorce, economic pressure, chronic illness, eviction, unwanted pregnancy) that are likely to happen throughout their lives. Each item was rated on a categorical scale (no/yes occurrence) and its emotional impact on the participant was scored on a 3-point Likert scale (0 = no occurrence; 1 = little impact; 3 = very high impact). The total emotional impact was obtained by a cumulative scoring of the reported intensity of the events suffered.

The Childhood Trauma Questionnaire-Short Form [38,39] was used to evaluate the personal history of abuse and neglect. It consists of 28 items with a 5-point Likert scale (1 = never; 5 = always) and five subtypes: physical neglect (α = 0.71), emotional abuse (α = 0.92), physical abuse (α = 0.88), sexual abuse (α = 0.94) and emotional neglect (α = 0.93) for our sample. The total score for each subscale was calculated by adding the score for each item on the corresponding subscale.

The Mini International Neuropsychiatric Interview [40] assesses on a categorical scale (no/yes) symptoms of the 16 most common psychiatric disorders in DSM-IV and ICD-10. Psychiatric scores obtained for each disorder correspond to a cumulative scoring of symptoms and not to a categorical diagnosis classification. None of the mothers in either group was under medication for psychiatric disorders at the time of testing. To obtain a global score, we submitted the psychopathological variables to a Principal Component Analysis except for Anorexia and Bulimia with zero scores (Major Depressive Episode, Dysthymia, Hypo/Manic Episode, Suicidality, General Panic Disorder, Agoraphobia, Social Phobia, Obsessive-Compulsive Disorder, Post-traumatic Stress Disorder, Alcohol Dependence/Abuse, Drug Dependence/Abuse, Psychotic Disorders, Generalized Anxiety Disorder and Antisocial Personality). Results gave a one-factor solution, Psychiatric Disorders, with high inter-correlations among the variables, KMO = 0.76, Eigenvalue = 4.82 and an explained variance of 72%.

The Interpersonal Reactivity Index [41,42] consists of 28 items with a 5-point Likert scale (1 = never; 5 = always) distributed into the following four scales: Empathic Concern Scale with feelings of warmth and concern for others (α = 0.60); Personal Distress Scale with feelings of anxiety and discomfort in interpersonal settings (α = 0.77); Perspective-Taking Scale to adopt the psychological point of view of others (α = 0.77); and Fantasy Scale to identify with fictional characters (α = 0.69), all for our sample. The total score for each subscale was calculated by adding the score for the corresponding items.

### 2.3. Procedure

Social workers reported on the participants’ family characteristics and asked mothers for permission to be contacted by phone. Those mothers who gave permission were informed about the study and the procedure upon their written acceptance. We avoid the use of the term “neglect” in the contact communications. Then, the collaborator visited them at their homes, collected the mother’s responses to the questionnaires, and collected the saliva sample. The collaborator added a preservative solution to the saliva samples using a Real Saliva DNA Sample Collection Kit (RBMSAL01, Real Laboratory, Valencia, Spain) according to the manufacturer’s instructions. Monetary compensation of EUR 50 was given to the mothers at the end of the session.

### 2.4. DNAm Assay and Methylation Analyses

The salivary samples were stored in the Microbiology lab placed at The University Hospital N.S. de Candelaria (Tenerife, Spain). DNA was extracted from the saliva samples using the Maxwell extraction kit (Maxwell^®^ 16 Buccal Swab LEV DNA Purification Kit- Cat.#AS1295, Promega Corporation, Madison, WI, USA). Concentration and purity of DNA was assessed using spectrophotometry. Quality assessment of DNA samples was performed with the TapeStation instrument. Library preparation and methylation sequencing were conducted at the University of Michigan Epigenomics Core in Ann Arbor (Ann Arbor, MI, USA). In short, 250 ng of sample DNA was bisulfite converted with the Zymo kits (Zymo Research, Orange, CA, USA) using the manufacturer’s incubation parameters specific for Illumina methylation arrays. The cleaned-up samples were then hybridized to the Infinium Methylation EPIC Bead Chip arrays, which measures methylation levels interrogating over 850,000 CpG loci per sample at single-nucleotide resolution. Snakemake was used to treat bioinformatics workflow [43]. Raw red/green IDAT files were read into R using the Bioconductor minfi package [44] (v1, 30.0). ENmix Bioconductor package [45] (v1.21.6), based on detection *p*-values and signal intensity, was used for the initial quality control. Two control participants and 13,901 probes were removed from the study for this reason. In addition, 50,229 probes were also removed by their proximity to annotated common Single Nucleotide Polymorphism (SNPs). After filtering samples and probes, a series of corrections was carried out using out of band Infinium I intensities to correct probe intensities and RELIC algorithm [45] for correcting the two dyes. Normalization between arrays was carried out by separate quantile normalizing of the methylated and unmethylated intensities for the Infinium I or II probes [45]. Probe-type biases were corrected with the beta-mixture quantile normalization method (BMIQ) [46]. The data were later corrected for possible batch effect arising from the samples run in different plates. Given that DNA derived from saliva shows cellular heterogeneity, DNA methylation levels were estimated for the sample and the relative proportion of epithelial cells and immune cells to be included as covariates in the analyses of epigenetic age. The value of the two variables (epithelial cells and immune cells) were calculated using the estimate LC function from EWAS tool R-package [47], which uses the houseman algorithm to estimate cellular proportions.

### 2.5. DNAm PhenoAge Clock

Epigenetic age was calculated from the DNA methylation levels of the 513 CpGs from the Illumina HM450 microarray data relevant for the PhenoAge clock, hereafter PhenoAge [23]. Although PhenoAge was developed using samples from whole blood, it strongly correlates with chronological age in a host of different tissues, such as saliva cells (*r* = 0.81) [23]. In general, the discrepancy reflected by the DNAm clock between an individual’s biological and chronological ages is called epigenetic acceleration age (EAA). It is measured in the following two ways: (a) differential scores between DNAm age and chronological age; and (b) residual scores obtained by regressing DNAm age on chronological age, that also represent both positive and negative deviations of the epigenetic age from chronological age. As both measures (differential and residual) are highly correlated, either can be used. For brevity, here we present only the results with the residual score and in the Supplementary Tables S1–S3, Figures S1 and S2 we show those with the differential scores that are equivalent to those of residuals. To prevent possible biological confounds, residual scores were corrected for the concentration of epithelial cells and immune cells [48], as well as for ancestry, given its differential distribution in the two groups of mothers (see Table 1). To prevent technical artefacts the experimental position of the samples over the plates was also included as a covariate [49]. Consequently, the final residual EAA scores were obtained regressing DNAm age on chronological age, correcting for the four variables mentioned.

### 2.6. Statistical Analyses

We compared the mean in EAA residual scores between the two groups using a two-sample t-test statistic. However, epigenetic age is sensitive to different factors traditionally associated with EAA, namely childhood maltreatment, adverse life events, psychiatric disorders and sociodemographic variables also measured in our sample. Thus, a multivariate approach was necessary to test the hypothesis on differences in epigenetic age acceleration between NG and CG groups. An ANCOVA model was used to examine the contribution of these covariates to EAA. The technical and biological variables were not included at this point because they had already been corrected when the EAA residual score was calculated. To prevent a high dimensionality of the model considering the size of the sample, two data-driven criteria were added to the theoretical foundation to select the variables to be included in the model. The first criterion was that only those variables showing group differences, or otherwise, showing significant relations to EAA, would be included as covariates. Thus, we performed *t*-test, *χ*^2^ and Pearson’s *r* correlations on the set of sociodemographic and psychological variables. As for the second criterion, only those variables showing non-collinearity effects between them would be included in the ANCOVA.

Finally, an ANCOVA analysis was performed to test the difference between the two groups in EAA, and the role of empathy, altogether with the relevant variables that met the inclusion criteria in the previous analysis. We also included interactions of all the study variables with the group to test for a possible specific effect for the NG. Once the ANCOVA was performed, all those variables not contributing to EAA were removed from the equation in two steps. A final ANCOVA was performed, only containing the variables contributing to EAA. Data were analyzed using R [50].

## 3. Results

### 3.1. Epigenetic Age Acceleration (EAA) in Neglect and Control Groups

**Figure 1:**
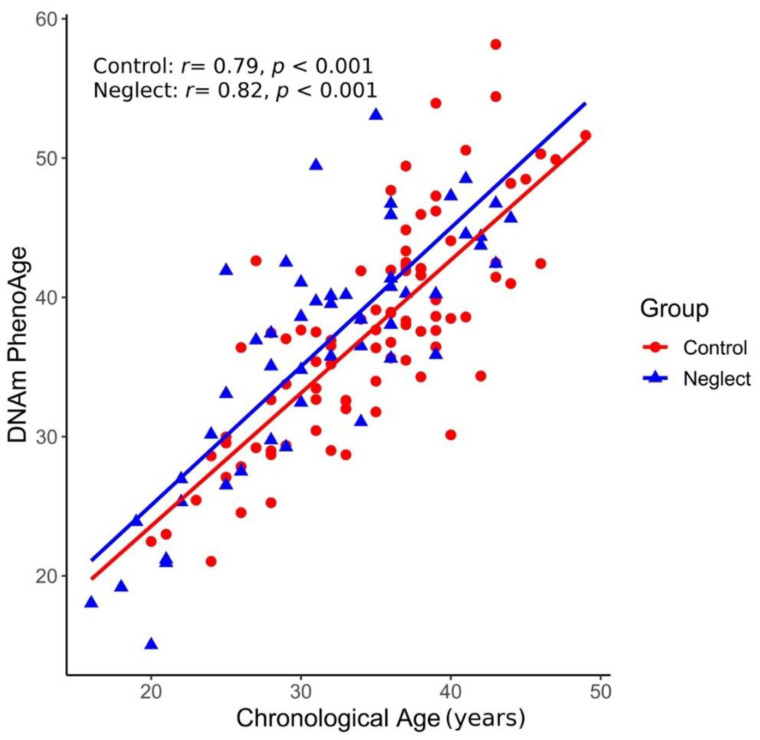
Relationships between epigenetic age and chronological age in Control and Neglect Groups. The scatterplot illustrates the significant and positive correlations between PhenoAge and chronological age (in years) for each group (red color corresponds to mothers and estimated regression lines in the Control Group and blue color corresponds to mothers and estimated regression lines in the Neglect Group).

We first explored the relation of PhenoAge with chronological age. Figure 1 presents the scatterplot of both variables by group. A significant positive relation spanning around twenty to fifty years of age was observed between the two variables in both groups of mothers (*r* = 0.79 and *r* = 0.82, *p* < 0.001 for the control and neglect groups, respectively). Both regression lines depicted a similar pattern of increases in epigenetic age with chronological age.

Then, we tested the differences in EAA between the groups. The mothers in the NG showed significantly higher mean scores in epigenetic age acceleration than the mothers in the CG for the residual score (NG: *M* = 1.21; *SD* = 4.87; CG: *M* = −0.70; *SD* = 4.05; *t*(135) = 2.46, *p* = 0.05, δ = 0.44). The results indicate higher age acceleration for the mothers in the NG compared to those in the CG.

### 3.2. Selection of Variables for the ANCOVA Model

Our first selection criterion established that only the variables showing significant group differences or otherwise with significant relationships to EAA should be entered in the ANCOVA model. A sociodemographic profile (Table 1) showed a lower maternal age, a higher number of pregnancies, a lower level of education and a higher percentage of mothers living in one-parent families and receiving financial assistance in the NG compared to the CG, all to be included in the model. Table 2 presents the group comparisons of psychological variables (top). The mothers in the NG perceived a significantly higher intensity in the adverse events suffered, were more likely to have had childhood maltreatment and were more vulnerable to psychiatric disorders than the mothers in the control group, all to be included in the model. No between-group differences were found for empathy factors. The corresponding correlations of empathy factors with EAA were performed for each group to test their possible contribution to the ANCOVA model (Table 2 bottom) shows that empathic concern was related to EAA in both groups.

Our second selection criterion established that all the selected variables should not show collinearity effects between them. None of them showed collinearity effects.

### 3.3. Group, Empathic Concern and Covariate Effects on EAA

Finally, we performed an ANCOVA to test group, empathic concern and sociodemographic and psychological variable effects on the criterion variable, EAA. The predictor variables were grouped (two levels) as factors, and as covariates: empathic concern (mean score); the sociodemographic variables: maternal age (mean score), number of pregnancies, type of family (dichotomized 0–1), level of education (1–3 scale) and financial assistance (dichotomized 0–1); the psychological variables: intensity of negative events (sum), childhood maltreatment (sum) and psychiatric disorder (factor score). The interactions between the group and the covariates were also modeled.

Once the initial ANCOVA had been performed, those covariates and/or their interactions with group, not showing a significant influence on EAA were removed until a solution was obtained only containing variables with significant effects (see Table 3). The results of the significant solution showed group differences and the contribution of empathic concern, education level, family type and the effect of their respective interactions by group on EAA.

**Figure 2:**
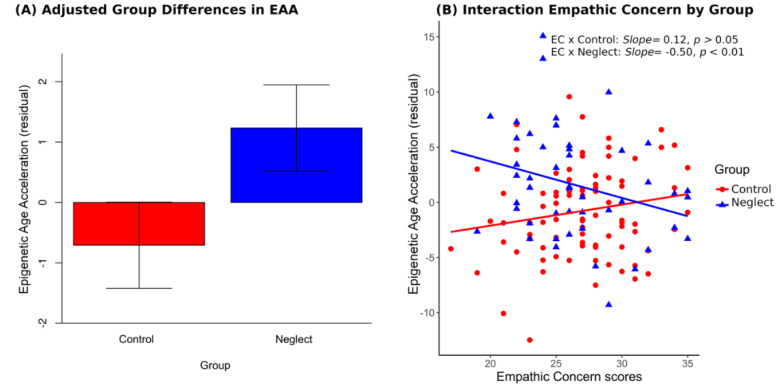
(**A**) Comparison of means showing higher epigenetic age acceleration (EAA) in NG compared to CG, once adjusted by empathic concern, educational level, family type and having discarded the contribution of maternal age, number of pregnancies, financial assistance, intensity of adverse events, childhood maltreatment and psychiatric disorders. (**B**) Empathic Concern interaction showed negative relationships with epigenetic age acceleration in NG only. An increase in empathic concern was associated with decreased epigenetic age acceleration for mothers in the neglect group (NG). For visual clarity, slopes for empathic concern were plotted from independent estimations and not from partial betas or adjusted by the rest of the variables.

Confirming our main hypothesis, results showed a main effect of group, indicating that mothers in the NG had higher EAA scores than the control mothers, *F*(1126) = 4.21, *p* < 0.05, (see Figure 2A), once adjusted by the rest of the covariates.

The adjusted effect of group on EAA also qualified the contributions of empathic concern, educational level and family type on EAA, evidenced by its interaction with them. Regarding empathic concern (see Figure 2B), the slope computation was significant and negative for NG (−0.39). This value resulted from subtracting the slope for empathy in the control group (*B* estimate = 0.19, *p* = 0.09) from the original slope for empathy in the neglect group (*B* estimate = −0.58, *p* = 0.002). A final *B* = −0.39 indicates that the higher the empathy is, the less the epigenetic age acceleration is only for the NG, *t*(126) = −3.04, *p* < 0.01, in line with our predictions.

**Figure 3:**
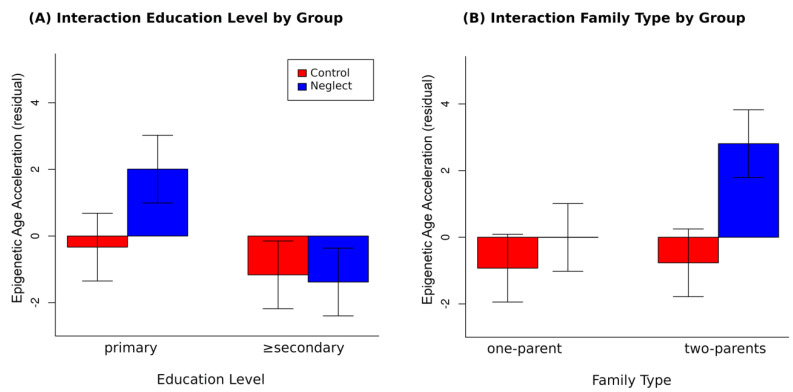
(**A**) Interactive effect of Level of Education and Group showing that EAA increases are obtained for those mothers with primary education in the NG only. In contrast, group differences were not significant at the secondary level. (**B**) Interactive effects of Type of Family and group showing that EAA increases are obtained for those mothers living in two-parent families in the NG only. In contrast, group differences were not significant in one-parent families.

As for the interaction effect of educational level by group, a similar computation as for empathic concern was made for the final slope (Figure 3A). The negative slope (*B* = −4.47) for the mothers in the NG showed that the higher the level of education is, the less the epigenetic age acceleration is, *F*(1126) = 5.46, *p* < 0.05, with non-significant effects in the CG (*B* = −0.82, *p* = 0.36), supporting the expected protective effect of maternal education.

The interaction effect of the type of family by group involved a different computation since it is a dummy variable. For the control mothers, the change from living in a one-parent family to living in a two-parent family did not modify the EAA (*p* = 0.65). However, the mothers in the NG living in the one-parent families differ in the EAA by 3.76 units from the mothers living in a two-parent family (Figure 3B). Thus, living in a two-parent family significantly increased the value of epigenetic age acceleration, *t*(126) = 2.27, *p* < 0.05, which seems at odds with what would be expected.

## 4. Discussion

This study examined the differences in EAA, based on PhenoAge, associated with extreme variations of maternal caregiving and the potentially protective role of empathy. The results showed that EAA was accelerated by more than two years (2.17 years in the differential EAA score) in those mothers who neglect their child as compared to mothers in the control group. This EAA was observed even though mothers in the NG were chronologically younger than in the CG on average and after adjusting for the potential contribution of different sociodemographic, psychological, technical and biological variables.

Evidence is still sparse for the effect of psychosocial risk and protective factors on adult variations in the EAA based on PhenoAge, mainly in relation to the impact of income, education, physical conditions and lifestyles [23]. This evidence is practically non-existent in the case of mothers. To better understand the factors that lead to group differences in the EAA, we tested a wide range of psychosocial risk factors included in the NG profile, and the contribution of trait empathy as a positive psychological factor. The risk profile obtained for the mothers in the NG in comparison to the control mothers showed that they are mostly younger, with a higher number of pregnancies, with half of them living in two-parent families and that they have a lower education level, suffer more economic difficulties and experience greater intensity of adverse events, greater childhood maltreatment and greater propensity for psychiatric disorders, in line with previous evidence [6,9,10,51].

Regarding empathy, we selected empathic concern as it was the only factor related to epigenetic age acceleration. Interestingly, empathic concern had also been the sole empathy factor related to the neural basis of neglectful caregiving in a previous study [34]. We hypothesized that empathic concern is a positive factor associated with reductions in EAA in the mothers with neglectful caregiving versus the control mothers. The results of the adjusted ANCOVA solution confirmed the positive EAA effect in the mothers of the neglect group versus the control. This deceleration effect was observed for the mothers in the NG within a range of scores in empathic concern similar to that of the mothers in the control group, given the absence of significant group differences.

Our finding on the protective role, in the sense of specificity, of empathic concern in EAA in neglectful caregiving sheds light on the relevance of affective versus cognitive components of empathy in epigenetic acceleration. Two recent gene-targeted epigenetic studies have addressed the modification of DNAm in relation to another affective component of empathy (personal distress) also in the context of dysfunctional caregiving. In the first study, dynamic DNAm trajectories of the oxytocin gene (OXT-promoter region) via blood samples predicted maternal intrusiveness at six months postpartum, with higher OXT DNAm in late pregnancy in intrusive compared to non-intrusive mothers [52]. The second DNAm study quantified the maternal OXT methylation via saliva samples and examined its relationship to the factors of trait empathy in the mothers, showing a positive correlation between oxytocin gene methylation and personal distress [53]. Intrusive behaviors and expression of anger and frustration have also been observed in parents with high personal distress [54]. In contrast, high empathic concern was more related to the mothers’ intention to provide care to the child’s needs [55] and the expression of warmth and positive affect [56]. Thus, the evidence suggests the relevance of affective empathy in both the OXT gene and the PhenoAge clock. However, according to its remarkable impact on caring behavior, empathic concern appears to play a more positive protective role and personal distress a negative role.

In our study, the negative relationship of empathic concern with EAA was specific to the mothers with neglectful caregiving, which is characteristic of a protective factor in Rutter’s classical sense in terms of specificity [14]. A relevant question arises about why this empathic capacity may be associated epigenetic aging deceleration in the NG only. Mothers who are more empathically apt and concerned about the others’ expression of emotions, even when carrying a heavy mental burden linked to life adversities and psychopathologies, would be better able to create firmer and more stable social bonds with other non-kin individuals. This proposal aligns with the evolutionary role of empathy [32]. Evidence has shown that empathic individuals are more able to help and receive emotional and social support in difficult situations, promoting prosocial actions [57]. They also feel more positive in social interactions [58] and used more adaptive coping strategies to deal with adversity [59]. In turn, better social functioning has a buffering effect against stress, promoting physical and mental health [33], which eventually may translate into a relative epigenetic age deceleration in our mothers. In addition, mothers with better social functioning would also be better able to attract informal and formal support providers who could help them cope with their role as caregivers.

In addition to empathy, the multivariate analysis also accounted for potential effects of risk profile factors, especially in the NG. Some of these risk factors might also act as potential candidates contributing to the empathy-EAA association. Among all the factors tested, only the mother’s education and family composition (one or two parents), both variables in interaction with the group, survived in the ANCOVA solution. We found that the higher the level of education is, the lower the epigenetic aging rate in mothers with neglectful caregiving is, with no significant trend in the case of the control mothers. The protective deceleration effect of higher education in DNA methylation levels is strongly supported by studies in large adult samples using PhenoAge [23,60], and Horvath and Hannum epigenetic clocks [16,26]. Educational failure is one of the long-term risks of having suffered childhood abuse and neglect, which is a factor more likely to occur in mothers who neglect their child [7].

Concerning family composition, unexpectedly, mothers living in two-parent families who neglect their child showed epigenetic age acceleration, with no significant trend in the case of the control mothers, most of whom lived with a partner. This is a new finding that deserves attention, since younger single mothers, with a lower income and a lower education level (a typical profile for our NG) are usually considered at risk of a lower quality of life, higher stress levels and poorer mental health [61]. However, there is longitudinal evidence that relationship quality is more important for the mental health of mothers and their children than the family type itself [62]. The study showed that living in a low-quality two-parent relationship has nearly the same negative effect as living as a single parent or in an unstable relationship. In our case, the results are even more extreme, in that they show that in mothers who neglect their children, living with a partner (50% of mothers) doubles the risk in terms of epigenetic age acceleration as compared to living alone. It is very likely that there will be more problems with the partner that transcends the children in mothers who neglect their child and who have also suffered childhood maltreatment and an adverse family life usually associated with adult insecure attachment styles [63]. These conditions may trigger the unmet need for affection that dominate the search for a partner over other considerations leading to poor or unstable intimate relationships [64,65]. Therefore, for those mothers, living in two-parent families can be accompanied by high interpersonal stress that affects the pace of biological aging, as a putative mediator of the effects of the psychosocial environment on health and disease [66].

Our findings should be considered in the context of several limitations. First, the cross-sectional design does not allow us to assess causal relations among risk and protective factors and epigenetic aging. Future studies should use causal models, post-intervention studies, longitudinal analysis or at least advance potential moderators/mediators of those associations to speak of causality legitimately. Second, given the selective nature of our research focused on a specific population, the sample is relatively small but rigorously based on external professional criteria. Third, the neglect cases and comparisons in this study were predominantly from mothers in lower socioeconomic strata, as is usually the case in public healthcare and social services, and these findings may not be generalizable to cases of neglect from middle- or upper-income families. However, socioeconomic support did not affect EAA in the final model. Fourth, as a general limitation in studies using epigenetic clocks for which the underlying biological mechanisms are not well established, we did not conduct a genome-wide analysis to elucidate the genetic determinants of differential epigenetic aging indexed by PhenoAge. Finally, we cannot exclude the possibility that non-significant contributions of some psychosocial risk factors might be due to limitations of PhenoAge in capturing methylation changes of those sites relevant to these factors.

## 5. Conclusions

The present study provides new evidence on epigenetic age acceleration in those mothers who neglect their children, applying the PhenoAge biomarker to the study of DNA methylation. We also reveal that empathy is associated with a reduction in EAA, only found explicitly in the mothers in the neglect group, pointing to a new facet of the evolutionary value of affective empathy. This facet represents the “sunny side” of protective psychological factors as opportunities to offset the well-documented “dark side” of psychosocial risk factors on epigenetic aging. Both findings, though preliminary, help expand our understanding of the neurobiology of parenting by opening the search for protective factors of epigenetic aging associated with variations in caregiver behavior. Promoting empathy, raising the mothers’ education level and providing personal support to help them avoid or abandon negative relationships with a partner can be positive for improving parental behavior. In addition, it can also provide a preventive strategy to reduce adult health vulnerabilities and poor social functioning that characterize the profile of these mothers. Downplaying these critical aspects of the caregiver role can frustrate or reduce the impact of intervention efforts focused primarily on the neglected child’s safety and wellbeing. Epigenetic factors may also account for the stability of the effects of parenting on offspring’s DNAm and developmental outcomes and the transmission of parenting behavior from one generation to the next for non-genomic processes, opening new lines for research and intervention to break the intergenerational cycle of maltreatment.

## Figures and Tables

**Table 1:** Sociodemographic profile in Control and Neglect Groups and biological variables.

	Control Group (*n* = 87) *M* (*SD*) or %	Neglect Group (*n* = 50) *M* (*SD*) or %	*t*(135)/*χ*^2^
Mean age of mother	34.72 (6.37)	31.36 (7.28)	2.82 **
Number of pregnancies	1.66 (0.73)	2.48 (1.3)	−4.13 ***
Mean age of the target child	3.67 (2.11)	3.59 (2.56)	0.20
Two-parent family %	72	50	6.0 *
Level of education (%)			16.62 ***
Primary school	43	80	
≥Secondary school	57	20	
Rural areas (%)	26	44	3.68
Unemployment (%)	58	70	1.31
Financial assistance %	24	68	23.63 ***
Ancestry of mother %			7.8 *
African	0	0.02	
European	0.88	0.98	
Latin American	0.12	0	
Immune cells (proportion)	1.09 (0.09)	1.12 (0.06)	−1.65
Epithelial cells (proportion)	0.02 (0.08)	0.01 (0.05)	0.81

* *p* < 0.05; ** *p* < 0.01; *** *p* < 0.001. Note: *M*: mean score, *SD*: standard deviation; *t*: *t*-student statistic; *χ*^2^: Chi-Square statistic. Group comparisons with mean scores were performed with t statistic, while those with percentage values were performed with Chi-Square (*χ*^2^) statistic.

**Table 2:** Group comparisons of the psychological variables and correlations to epigenetic age acceleration (EAA).

Comparisons	Control Group (*n* = 87) *M* (*SD*)	Neglect Group (*n* = 50) *M* (*SD*)	*t*(135)	*δ*
Empathy				
Empathic concern	26.66 (4.26)	26.98 (3.79)	−0.45	0.08
Personal distress	18.54 (4.25)	18.08 (4.52)	0.58	0.10
Perspective taking	24.33 (4.80)	24.80 (3.90)	−0.61	0.11
Fantasy	20.3 (4.64)	21.3 (4.5)	−1.17	0.21
Intensity events	11.59 (7.70)	16,66 (8.70)	−3.52 ***	0.62
Child maltreatment	33.98 (11.25)	47.22 (21.37)	−4.06 ***	0.72
Psychiatric disorders	−0.26 (0.80)	0.40 (1.14)	−3.59 ***	0.64
**Correlations**	**EAA (*r*)**	**EAA (*r*)**		
Empathy				
Empathic concern	0.19 *	−0.27 *		
Personal distress	−0.20	0.03		
Perspective taking	0.05	0.12		
Fantasy	0.03	−0.12		

Note: *M*: mean score, *SD*: standard deviation; *t: t*-student statistic; *δ*: delta * *p* < 0.05, *** *p* < 0.001.

**Table 3:** Effects of Group, Empathic concern, Family type, Educational level and their interaction with Group on EAA in the final ANCOVA solution ^a^.

Variables	*F*(1126)	*p* Value
Empathic concern	0.06	0.80
Family type	5.28	0.02
Educational level	5.17	0.02
Group	4.21	0.04
Empathic concern × Group	9.25	0.00
Family type × Group	5.17	0.02
Educational level × Group	5.46	0.02

Note: *F*: *F* statistic. ^a^ The solution was obtained after discarding the non-significant contributions of maternal age, number of pregnancies, financial assistance, intensity of adverse events, childhood maltreatment and psychiatric disorders.

## Data Availability

Data that supports the findings of this study are available on request from the corresponding author.

## Supplementary Materials

The following are available online at https://www.mdpi.com/article/10.3390/brainsci11111376/s1, Table S1: Mean group comparison of epigenetic age acceleration for the differential measure, Table S2: Correlations of psychological and biological variables with epigenetic age acceleration (differential EAA), Table S3: Results for the initial model of ANCOVA with all study variables that meet the selection criteria on differential EAA scores, Figure S1: Comparison of EAA means in NG and CG and the interaction between Empathic Concern and differential EAA, Figure S2: Interaction effects of Level of Education (A) and Type of Family (B) and Group.
