# Supplementary material for: Reduction in Epigenetic Age Acceleration Is Related to Empathy in Mothers with Neglectful Caregiving
Source: Brain Sci. 2021 Oct 21;11(11):1376. doi: 10.3390/brainsci11111376 (PMC8615407; doi:10.3390/brainsci11111376)
Supplement: Supplementary file 1 [file brainsci-11-01376-s001.zip › brainsci-1373209-supplementary.pdf]

Epigenetic age acceleration and the protective role of empathy in mothers with neglectful caregiving

Supplementary

Table S1. Mean group comparison of epigenetic age acceleration for the residual measure

EAA	**Control Group (n = 87)** *M (SD)*	**Neglect Group (n =50)** *M (SD)*	**Comparison** ***t*(135)**	**Effect size** *δ*
Residual	-0.51 (4.8)	1.24 (5.1)	-1.99*	0.35

*$p<.0.05$

Table S2. Correlations of psychological and biological variables with epigenetic age acceleration (residual EAA)

Correlations	**EAA (*r*)**	**EAA (*r*)**
Empathic concern	0.24*	-0.30*
Personal distress	-0.11	0.08
Perspective taking	0.08	0.10
Fantasy	-0.02	-0.17
Leukocytes	-0.51***	-0.18
Epithelial cells	0.53***	0.33*

*$p < 0.05$, ***$p < 0.001$

Table S3. Estimate values of Empathic concern, Family characteristics, and Epithelial cell covariates by group in the final ANCOVA solution on epigenetic age acceleration (residual EAA).

ANCOVA solution[a]

Variables	Estimate	Std. Error	*t value*
Intercept	-0.66	0.47	-1.40
Group (NG and CG)	1.66	0.86	1.93*
Empathic Concern	0.18	0.12	1.48
Education level	-0.06	0.78	-0.07
Family type	0.23	1.02	0.23
Epithelial cell	32.36	5.55	5.83***
Empathy x NG	-0.54	0.19	-2.77**
Education level x NG	-3.73	1.59	-2.33*
Family type x NG	3.35	1.63	2.05*

*$p < 0.05$, **$p < 0.01$, ***$p < 0.001$

[a]The final solution was obtained once discarded the non-significant contributions of maternal age, number of pregnancies, financial assistance, intensity of negative events, childhood maltreatment and psychiatric disorders, as well as plate position, as a nuisance variable.

(A) Adjusted Group Differences in EAA (B) Interaction Empathic Concern by Group

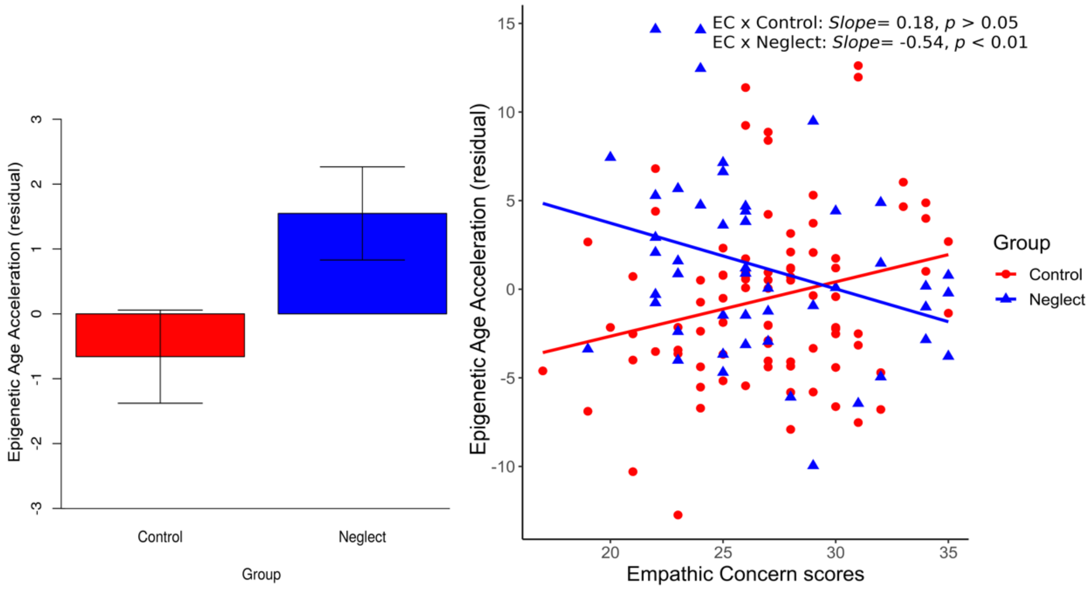

Figure S1. (A) Mean comparisons showing higher epigenetic age acceleration (EAA) in NG compared to CG, once adjusted for contributions of maternal age, number of pregnancies, financial assistance, intensity of negative events, childhood maltreatment and psychiatric disorders. (B) Empathic Concern interaction showing protective effects on epigenetic age acceleration in NG only. An increase in empathic concern was associated with a decrease in epigenetic age acceleration for mothers in the neglect group (NG) only. For graphical clarity, slopes for empathic concern were plotted from independent estimations and not from partial betas or adjusted by the rest of the variables.
